# Acromion-axillary nerve distance and its relation to the arm length in the prediction of the axillary nerve position: a clinical study

**DOI:** 10.1186/s13018-022-03085-6

**Published:** 2022-04-24

**Authors:** Cem Yildirim, Mehmet Demirel, Erhan Bayram, Mehmet Ekinci, Murat Yılmaz

**Affiliations:** 1 Department of Orthopaedics and Traumatology, Başakşehir Çam ve Sakura City Hospital, University of Health Sciences, Istanbul, Turkey; 2 Department of Orthopedics and Traumatology, İstanbul School of Medicine, Istanbul University, Istanbul, Turkey; 3 Department of Orthopaedics and Traumatology, Gaziosmanpaşa Medicalpark Hospital, Istinye University, Istanbul, Turkey; 4 Department of Orthopaedics and Traumatology, Haseki Training and Research Hospital, University of Health Sciences, Istanbul, Turkey

**Keywords:** Axillary nerve, Trans-deltoid approach, Deltoid-splitting approach, Iatrogenic nerve injury, Safe zone

## Abstract

**Background:**

Because of the broad anatomic variation in the course of the axillary nerve, several cadaveric studies have investigated the acromion-axillary nerve distance and its association with the humeral length to predict the axillary nerve location. This study aimed to analyze the acromion-axillary nerve distance (AAND) and its relation to the arm length (AL) in patients who underwent internal plate fixation for proximal humerus fractures.

**Methods:**

The present prospective study involved 37 patients (15 female, 22 male; the mean age = 51 years, age range 19–76) with displaced proximal humerus fractures treated by open reduction and internal fixation. After anatomic reduction and fixation were achieved, the following parameters were measured in each patient before wound closure without making an extra incision or dissection: (1) the distance from the anterolateral edge of the acromion to the course of the axillary nerve was recorded as the acromion-axillary nerve distance and (2) the distance from the anterolateral edge of the acromion to the lateral epicondyle of the humerus was recorded as arm length. The ratio of AAND to AL was then calculated and recorded as the axillary nerve index (ANI).

**Results:**

The mean AAND was 6 ± 0.36 cm (range 5.5–6.6), and the mean arm length was 32.91 ± 2.9 cm (range 24–38). The mean axillary nerve ratio was 0.18 ± 0.02 (range 0.16 to 0.23). There was a significant moderate positive correlation between AL and AAND (*p* = 0.006; *r* = 0.447). The axillary nerve location was predictable in only 18% of the patients.

**Conclusion:**

During the anterolateral deltoid-splitting approach to the shoulder joint, 5.5 cm from the anterolateral edge of the acromion could be considered a safe zone to prevent possible axillary nerve injury.

## Background

In the operative treatment of proximal humerus fractures, the deltopectoral approach is the most widely used approach for internal plate fixation. However, this traditional approach offers limited access to the posterolateral aspect of the proximal humerus that may make reduction and fixation of a displaced greater tuberosity fragment and proper plate placement difficult [1]. Alternatively, with minimal soft-tissue dissection, the anterolateral deltoid-splitting approach can provide direct access and excellent visualization of the greater tuberosity and the site of the plate placement [2, 3]. However, there is an increased risk for axillary nerve injury when using the deltoid-splitting approach [4, 5].

Although it is generally accepted that the axillary nerve crosses the humerus horizontally nearly 50 mm distal to the acromion in clinical practice, various anatomic studies have defined a broad range of safe zones for deltoid-splitting approaches, varying from 30 to 70 mm distally to the acromion [6–9]. Furthermore, it has been shown that a safe zone for a nerve may change in size as per the extremity length [10]. Because of the large anatomic variation in the course of the axillary nerve from one individual to another, several cadaveric studies have explored the acromion-axillary nerve distance and its association with the humeral length to predict the axillary nerve location [11–14]. Nonetheless, to the best of our knowledge, no clinical study has not yet been conducted to investigate the relationship between location of the axillary nerve and humeral length to date.

This study aimed to analyze the acromion-axillary nerve distance (AAND) and its relation to arm length (AL) in patients who underwent internal plate fixation for proximal humerus fractures. The authors hypothesized that AAND has a significant correlation with the humeral length and can be used to predict the axillary nerve location during the anterolateral deltoid-splitting approach.

## Methods

The present prospective study involved 37 patients with displaced proximal humerus fractures who were treated by open reduction and internal fixation at a single tertiary trauma referral center from January 2017 to May 2019. Inclusion criteria were patients aged > 18 years, with proximal humerus fractures without a previous history of shoulder surgery. Exclusion criteria were patients with polytrauma, pathological fracture, concomitant fracture of the same upper extremity, limb discrepancy, or congenital deformity. Informed consent was obtained from all the patients preoperatively; ethical approval was obtained from the institutional ethical committee (88-2021, 06.10.2021).

### Operative technique

All surgical procedures were performed by a single experienced orthopedic trauma surgeon within a week of the injury, using the anterolateral deltoid-splitting approach. All the operations were performed under general anesthesia. The patients were placed in a beach-chair position, and bony landmarks were marked before making the incision. A longitudinal incision was made from the anterolateral edge of the acromion, which extended distally along the long axis of the humerus, and dissection was performed between the anterior and middle thirds of the deltoid muscle fibers. The axillary nerve was then palpated and visualized carefully. After ensuring adequate protection of the axillary nerve, the dissection was extended distally. The exposed region of the shoulder was divided into two parts by the axillary nerve. While the superior part was used to reduce the fracture, the distal part was used to fix the plate to the humeral shaft. Later, the fracture was reduced, and Kirschner wires were inserted for temporary fixation. The anatomic proximal humerus plate (TST™, Locked Proximal Humerus Plate, Pendik, İstanbul, Turkey) was then placed under the axillary nerve, and the rotator cuff was repaired if required. The final position was checked using fluoroscopy. The wound was closed in layers, and a drain was inserted inside the subcutaneous tissue. Postoperatively, the arm was placed in a sling for controlled physical therapy.

### Outcome parameters

**Fig. 1:**
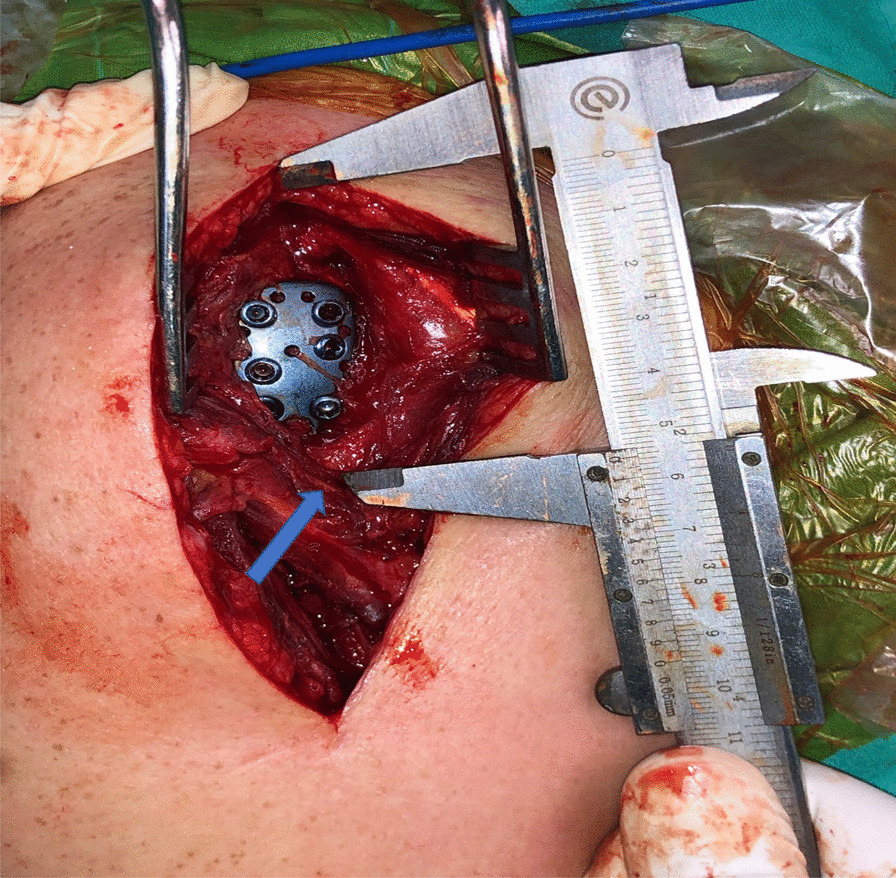
Representative figure showing the intraoperative measurement method of the distance between the anterolateral edge of the acromion and the axillary nerve (blue arrow) using a caliper

**Fig. 2:**
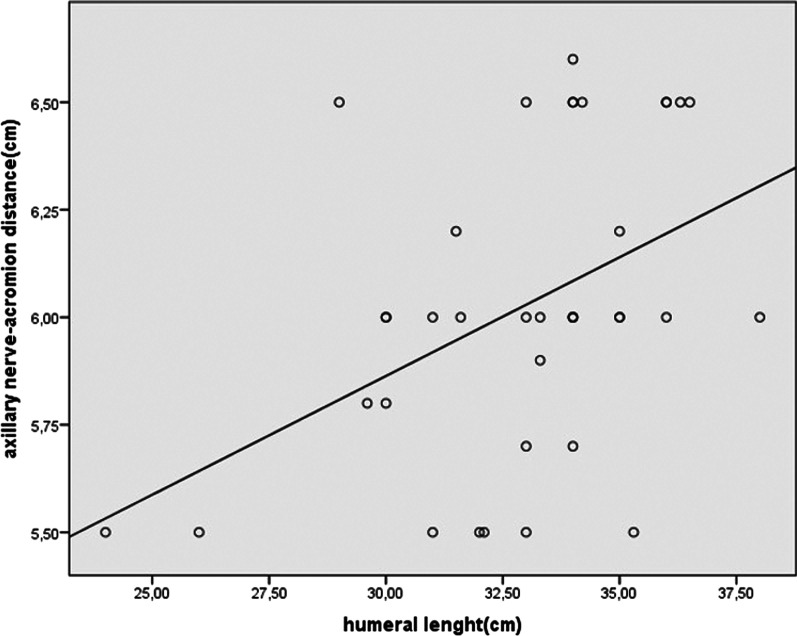
Graph illustrating the correlation between arm length and axillary nerve distance from the anterolateral edge of the acromion

Within the routine steps of the planned operation, after anatomic reduction and fixation were achieved, the following parameters were measured in each patient before wound closure without additional dissection through incision: (1) the distance from the anterolateral edge of the acromion to the course of the axillary nerve was recorded as the acromion-axillary nerve distance (Fig. 1), and (2) the distance from the anterolateral edge of the acromion to the lateral epicondyle of the humerus was recorded as AL [11]. Each parameter was measured with the arm positioned in 45° internal rotation and 30° abduction. The ratio of AAND to AL was then calculated and recorded as the axillary nerve index (ANI) for each patient as described by Çetik et al. [11]. Furthermore, the correlation between AAND and AL was investigated, and a subgroup analysis was performed as per the sex.

### Statistical analysis

All statistical analyses were performed using SPSS 25.0 software (SPSS Inc., Chicago, IL, USA). A *p* value of 0.05 was considered statistically significant. Descriptive statistics were given as mean, standard deviation, percent, lowest (min), and highest (max) values. The Kolmogorov–Smirnov test was used to verify the normal distribution of the variables. The correlation between AAND and AL was evaluated using the Spearman correlation analysis in a linear regression model. The level of correlation was interpreted according to the degree of relationship as strong (*r* = 0.7–1), moderate (*r* = 0.4–0.7), or low (*r* = 0.2–0.4) after taking significant correlation (*p* < 0.01 or *p* < 0.05) values into consideration [15, 16]. AAND was predicted using the formula obtained from linear regression analysis (AAND = 4.207 + 0.055 X AL). Goodman and Kruskal Tau values were used in the comparison of overestimated and underestimated values.

## Results

There were 37 patients included in the study: 15 females and 22 males with the mean age of 51 (range 19–76) years. According to the Neer classification system [17], there were 15 two-part (41%), 20 three-part (54%), and two four-part (5%) humerus fractures.

The mean AAND was 6 ± 0.36 cm (range 5.5–6.6 cm), and the mean AL was 32.9 ± 2.9 cm (range 24–38 cm). The mean axillary nerve index was 0.18 ± 0.02 (range 0.16–0.23) (Table 1). A significant moderate positive correlation was identified between AL and AAND (*p* = 0.006; *r* = 0.447) (Fig. 2). We were able to predict the location of the axillary nerve in 18% of the patients using the regression analysis. In subgroup analysis between males and females, there were no significant differences in AAND, AL, and axillary nerve index (*p* = 0.052, *p* = 0.09 and *p* = 0.988; respectively) (Table 2). Mean square error values were found similar as 0.0930 in women and 0.1082 in men. When overestimated and underestimated values were compared, no significant difference was found in terms of age (*p* = 0.691), gender (*p* = 0.141), and Neer fracture type (*p* = 0.478) parameters.

**Table 1:** Demographic data of the study participants

Number of patients	37
Age (years), mean	51 (range 17–76)
Gender (Male/Female)	22/15
AAND (cm), mean ± SD	6 ± 0.36 cm (range 5.5–6.6)
AL (cm), mean ± SD	32.91 ± 2.9 cm (range 24–38)
Axillary nerve index (AAND/AL), mean ± SD	0.18 ± 0.02 (0.16–0.23)

AAND, Acromion-axillary nerve distance; AL, Arm Length; SD, Standard Deviation

**Table 2:** Results of subgroup analysis as per gender

Variables	Group female	Group male	*p*
	(*n* = 15)	(*n* = 22)	
	Mean ± SD (range)	Mean ± SD (range)	
ANND (cm)	5.87 ± 0.35	6.12 ± 0.33	0.052
	(5.5–6.5)	(5.5–6.6)	
AL (cm)	31.90 ± 3.21	33.55 ± 2.49	0.090
	(24.0–36.0)	(29.0–38.0)	
ANI (AAND/AL)	0.18 ± 0.01	0.18 ± 0.01	0.711
	(0.17–0.23)	(0.16–0.22)	

AAND, Acromion-axillary nerve distance; AL, Arm Length; ANI, Axillary nerve index; SD, Standard Deviation

*p* < 0.05 was considered statistically significant

## Discussion

Although the anterolateral deltoid-splitting approach can ensure direct access and excellent visualization of the plating area in the management of proximal humerus fractures [2, 3], there is an increased risk for axillary nerve injury, which is the most common neurological complication associated with surgery of proximal humerus fractures [6, 18, 19]. Accordingly, defining the safe zone for the axillary nerve is essential to avoid iatrogenic injury. Various anatomic studies have defined a broad range of safe zones for deltoid-splitting approaches, varying from 30 to 70 mm distally to the acromion [6–9]. Because of the broad anatomic variation in the course of the axillary nerve, the AAND and its association with the AL were investigated to predict the axillary nerve location [11–14, 20, 21] in some cadaveric studies. Nonetheless, according to our literature review, the relationship between the axillary nerve location and AL has not been investigated in a clinical setting to date.

The present study aimed to describe a safe area for executing the anterolateral deltoid split approach during open reduction–plate fixation for managing patients with proximal humerus fractures. We found that AAND was 6.0 ± 0.36 cm, moderately correlated with AL, and ANND could be predicted according to AL in only 18% of the patients. In contrast, Chang-Meen et al. [14] found that in addition to the strong correlation between AAND and humeral length, the use of the ANI with the humeral length provided the shortest predictions of AAND, with a 97.8% probability of safety. The authors concluded that AL and ANI may be helpful for predicting the axillary nerve in clinical practice. This difference may be due to the prediction method used in Chang-Meen et al.'s study being different from ours. Although they used the shortest AAND to predict the axillary nerve location, we created a linear regression analysis formula based on AL in predicting axillary nerve location. Also, Chang-Meen et al. conducted measurements with the arm positioned at the side in neutral rotation, we did with the arm in 45° internal rotation and 30° abduction, as the humeral defect is best visualized in this position when using the trans-deltoid approach [22].

Numerous studies have attempted to measure AAND and found significant variations with a range of 4.5 to 7.5 cm [8, 11–13, 23, 24]. Kongcharoensombat et al. [12] calculated the mean distance of the axillary nerve from the anterolateral acromion as 6.39 cm (ranging from 4.6 to 8.2 cm), and Cetik et al. [11] found the distance of the axillary nerve from the anterolateral acromion to be 6.08 cm (ranging from 5.20 to 6.90 cm). Both previous studies observed a significant correlation between the distance of the axillary nerve from the anterolateral acromion and humeral length. In contrast to the cadaveric studies of Kongcharoensombat [12] and Cetik et al. [11], the present study was conducted in a clinical setting. All measurements were performed intraoperatively after the anatomic reduction and fixation were completed. In this regard, our study is advantageous over previous cadaveric studies in the literature.

While using the anterolateral approach for proximal humeral fractures, the plate should be inserted under the axillary nerve to dissect the nerve carefully, and potential injury could be prevented. Also, the shortest distance should be considered during dissection to minimize the risk of potential axillary nerve injury. We measured the minimum distance of the axillary nerve to be 5.5 cm from the acromion. Hence, this distance could be considered a safe zone according to the present study’s findings. In the study by Cetik et al. [11], this distance was measured as 5.2 cm. However, these data contradict the findings of Kongcharoensombat et al. [12] because the axillary nerve was found located at < 5 cm in 13% of the cadaver shoulders.

In our study, the calculated mean axillary nerve index was lower than that given by Cetik et al. [11] and Kongcharoensombat et al. [24]. The exact prediction ratio of the location of the axillary nerve according to the humeral length of the patients was 18%, which was lower than the expected value. Therefore, we believe it would be safer to use distance instead of the ratio.

Our study has several limitations. First, the number of patients who participated in the study was limited. Second, the measurements were made using a manual caliper, allowing for human errors. Third, all the measurements were performed after the anatomic reduction was completed. However, in case of deformity due to proximal humerus fracture before the reduction was performed during the exposure, this distance is likely to be shortened.

## Conclusions

Evidence from this study has demonstrated that during the anterolateral deltoid-splitting approach to the shoulder joint, 5.5 cm from the anterolateral edge of the acromion could be considered as a safe zone for the prevention of possible axillary nerve injury. Predicting the location of the axillary nerve using the AL was possible in only 18% of the patients; thus, it would be safer to use the distance of 5.5 cm instead of relying on the axillary nerve index.

## Data Availability

The data used and/or analyzed during the current study are available from the corresponding author or the first author on reasonable request.

## Abbreviations

AAND: Acromion-axillary nerve distance
AL: Arm length
